# Double domain polyethylenimine-based nanoparticles for integrin receptor mediated delivery of plasmid DNA

**DOI:** 10.1038/s41598-018-25277-z

**Published:** 2018-05-01

**Authors:** Hossein Sadeghpour, Bahman Khalvati, Elaheh Entezar-Almahdi, Narjes Savadi, Samira Hossaini Alhashemi, Mohammad Raoufi, Ali Dehshahri

**Affiliations:** 1 Pharmaceutical Sciences Research Center, Shiraz University of Medical Sciences, Shiraz, Iran; 2 Department of Medicinal Chemistry, School of Pharmacy, Shiraz University of Medical Sciences, Shiraz, Iran; 3 Medicinal Plants Research Center, Yasuj University of Medical Sciences, Yasuj, Iran; 4 Department of Pharmaceutical Biotechnology, School of Pharmacy, Shiraz University of Medical Sciences, Shiraz, Iran; 5 Department of Nanotechnology and Nanotechnology Research Center, Faculty of Pharmacy, Tehran University of Medical Sciences, Tehran, Iran

## Abstract

The objective of the present study is to conjugate L-thyroxine PEI derivative onto another PEI to compensate the amine content of the whole structure which has been utilized for the ligand conjugation. Since α_v_β_3_ integrin receptors are over-expressed on cancer cells and there is binding site for L-thyroxine on these receptors, PEI conjugation by L-thyroxine along with restoring the PEI amine content might be an efficient strategy for targeted delivery using polymeric nanoparticles. The results demonstrated the ability of the PEI conjugate in the formation of nanoparticles with the size of around 210 nm with higher buffering capacity. The conjugated PEI derivative increased the transfection efficiency in the cell lines over-expressing integrin by up to two folds higher than unmodified PEI, whereas in the cell lines lacking the integrin receptors there was no ligand conjugation-associated difference in gene transfer ability. The specificity of transfection demonstrated the delivery of plasmid DNA through integrin receptors. Also, the results of *in vivo* imaging of the polyplexes revealed that ^99m^Tc-labeled PEI/plasmid DNA complexes accumulated in kidney and bladder 4 h post injection. Therefore, this PEI derivative could be considered as an efficient targeted delivery system for plasmid DNA.

## Introduction

Immunotherapy has been considered as a new paradigm for the treatment of various types of cancers in recent years^1,2^. Several cytokine-based gene therapy approaches have been utilized against cancer including the application of Interleukin-12 (IL-12). There are some reports indicating the antitumor effect of this protein due to its ability to induce the secretion of interferon gamma (IFN-γ) as well as the activation of cytotoxic T lymphocytes (CTLs) and natural killer (NK) cells. In other words, IL-12 acts as a bridge between innate and adoptive immunity^3–5^. Despite the antiangiogenic effects of IL-12, its wide clinical applications have been limited not only for low *in vivo* efficacy but also for severe dose-dependent systemic toxicity^5^. Considering the deadly adverse reactions associated with the systemic administration of recombinant IL-12 protein in human body, researchers are seeking for alternative strategies including the transfer of IL-12 plasmid into the target tissue or organ. Since the elevated levels of IFN-γ has been considered as the major factor resulting in the systemic toxicity of IL-12, the production of the therapeutic protein inside the tumor site may reduce its systemic toxicity^6–9^. However, the successful transfer of such nucleic acid materials into the target cells has remained unfulfilled due to the lack of efficient, targeted, non-toxic and cost effective delivery systems^10,11^. Substantial progresses in the development of non-viral gene carriers and particularly the polymer-based gene delivery systems have opened up new horizons for the future advances of gene therapy^11^. Among the polymers used as gene carrier, polyethylenimine (PEI) has shown great potential for gene delivery due to its high amine content resulting in the significant proton sponge effect which consequently leads to the early escape of the complexes from endo/lysosomal compartments^12,13^. However, the relatively high toxic effects and lack of targeting have limited the broad clinical application of PEI. Therefore, the conjugation of targeting ligands via the amines on the surface of PEI has been proposed to direct the PEI/nucleic acid complexes into a specific site of action. These conjugation strategies may also reduce the cell-induced toxicity of PEI due the reduction of significant positive charge density on the polymer surface which is responsible for the induction of toxic effects^9,14–16^.

The targeted delivery of nucleic acid materials could be carried out via different receptors including integrin receptors^9,14^ as well as asialoglycoprotein^15,17,18^ folate^19^ and transferrin^20^. There is a great attention towards the application of vitronectin receptors which provide the connection between the cell signaling pathways and extracellular matrix^21^. The involvement of α_v_β_3_ integrin receptors in tumor metastasis and angiogenesis as well as the over-expression of the receptor on several cancer tissues including gliobalstoma, prostate carcinoma and more importantly on the vessels of tumors has made the receptor as a suitable candidate for targeted delivery of various therapeutic or diagnostic agents into the target cells^22^. The integrin receptor has been found to be recognized by tripeptide sequence arginine-glycine-aspartic acid (RGD)^23^. More interestingly, there are some studies reporting the existence of a recognition site for the thyroid hormone (L-thyroxine, T_4_) on the extracellular domain of the integrin receptors. It seems that this binding site is near or even at the RGD binding site^24^. These characteristics make L-thyroxine as a potential candidate for targeting through integrin receptors. Our previous investigation demonstrated the significant ability of the L-thyroxine and its deaminated analogue, tetraiodothyroaceticacid, for integrin receptor mediated delivery of plasmid DNA^9,14^. According to the results obtained in these studies, a double domain L-thyroxine conjugated PEI derivative was synthesized in the present study in which two PEI molecules were attached via a succinic acid linker. The unmodified PEI incorporated in the final conjugate was proposed to act as the delivery domain whereas the L-thyroxine conjugated PEI section was suggested to be the targeted domain. Since the simple conjugation of targeting ligands onto the PEI structure occurs through the primary amines on the surface of PEI molecules and these amines are necessary for the induction of proton sponge effect, it was hypothesized that the separation of targeting and delivery domains might be an effective strategy to benefit the advantages of the conjugation of targeting ligand on the PEI structure while maintaining its high primary amine content. There are some reports suggesting the preservation of primary amines or whole amine content of the polycationic compounds along with the conjugation of targeting moieties may enhance the ability of PEI based gene delivery systems^14^. The PEI/plasmid DNA complexes were characterized with respect to particle size, zeta potential, plasmid DNA binding affinity, protection of plasmid against enzymatic degradation as well as cytotoxicity and the ability to transfer IL-12 plasmid into OVCAR-3, an ovarian cancer cell line over-expressing integrin α_v_β_3_^25^, and compared with HepG2 cells lacking these receptors. Moreover, the radiolabeled complexes were injected to the mice and their biodistribution was studied.

## Materials and Methods

### Ethics statement

The present investigation was approved by Shiraz University of Medical Sciences. Also, all the methods used in this study were carried out in accordance with the approved guidelines and regulations and all the experimental protocols were approved by Shiraz University of Medical Sciences.

### Materials

Branched polyethylenimine (bPEI; average MW 25 kDa), succinic anhydride, N-hydroxy succinimide (NHS), 1-ethyl-3-[3-dimethylamino-propyl] carbodiimide hydrochloride (EDC), N-[2-hydroxyethyl] piperazine-N′-[2-ethanesulfonic acid] (HEPES) and 3-(4,5-dimethylthiazole-2-yl)-2,5-diphenyltetrazolium bromide (MTT) were purchased from Sigma-Aldrich (Munich, Germany). 3,5,3′,5′-tetraiodo-L-thyronine (L-thyroxine) was kindly donated by Iran Hormone Pharmaceutical Company (Tehran, Iran). Plasmid pUMVC3-hIL12 (human interleukin-12 under control of the cytomegalovirus enhancer/promoter) was obtained from Aldevron (Madison, WI, USA). Endofree Mega Plasmid Kit was purchased from Qiagen (Valencia, CA, USA). Human IL-12 (p70) ELISA Kit was obtained from BD Bioscience (Heidelberg, Germany). DNA ladder 1 kb and ethidium bromide (EtBr) were purchased from Cinnagen (Tehran, Iran). Fetal bovine serum (FBS), antibiotics and Roswell Park Memorial Institute Medium (RPMI 1640) were obtained from Gibco (Gaithersburg, MD, USA). Dialyses were carried out by Spectra/Por dialysis membranes (Spectrum Laboratories, Houston, TX, USA). All solvents were purchased from Sigma-Aldrich (Munich, Germany) and were of the highest purity available.

### Preparation of plasmid

Plasmid encoding human IL-12 (pUMVC3-hIL12) was transformed into *Escherichia coli* bacterial strain DH5α, incubated in Luria-Bertani (LB) media and then extracted and purified from the culture pellets using the Qiagen Endofree Mega Plasmid Kit according to the manufacturer’s instructions. The purity and the concentration of the plasmid were assessed using a UV–visible spectrometer (Shimadzu, Japan) at 260 and 280 nm.

### Synthesis of bPEI derivatives

The synthesis of double domain bPEI derivative was carried out in three steps. The preparation of succinated PEI was carried out as described in previous studies^26^. Briefly, desired amounts of succinic anhydride was added to the PEI solution and allowed to be proceed for 3 hours with shaking. Then the product dialyzed (10000 Da cut off Spectra/Por membrane) first against NaCl solution (0.25 M) and then twice against water to at 4 °C. Finally, the aqueous solution was lyophilized and the fluffy materials were characterized by ^1^H-NMR (D_2_O) spectroscopy using a Bruker Avance DRX-500 MHz NMR spectrometer (Bruker, Ettlingen, Germany)^26^.

The L-thyroxine conjugated bPEI was prepared as described earlier^14^. Briefly, 25 kDa branched PEI (0.25 g) was dissolved in water (5 ml) and mixed with the solution of L-thyroxine in water. In order to activate the carboxylic acid groups of L-thyroxine, 1 mole equivalents of N-hydroxy succinimide (NHS) and 1-ethyl-3-[3-dimethylamino-propyl] carbodiimide hydrochloride (EDC) were dissolved in water and added dropwise to the solution. Then the desired amount of L-thyroxine in water was added dropwise to the PEI solution with constant shaking and the reaction was carried out for 24 hours at room temperature. Finally, the crude product dialyzed (10000 Da cut-off Spectra/Por membrane) against water three times. Following the lyophilization process, the product was characterized by ^1^H-NMR (D_2_O) spectrometry.

In order to prepare the double domain derivative of bPEI, succinated PEI derivative was added dropwise to the solution of L-thyroxine conjugated bPEI over a period of 3 h followed by the addition of 1 mol equivalents of 1-ethyl-3-[3-dimethylaminopropyl] carbodiimide hydrochloride (EDC) and N-hydroxybenzotriazole to activate the carboxylate groups of succinated PEI for the formation of amide linkage between succinated PEI and the amines of L-thyroxine conjugated bPEI. The reaction mixture was allowed to proceed at room temperature for 24 h with constant shaking. The solution was dialyzed (25000 Da cut-off Spectra/Por membrane) against water three times followed by the lyophilization and the characterization by ^1^H-NMR (D_2_O) spectrometry. Also, the primary amine content of bPEI derivatives and the conjugation degrees were determined by 2,4,6-trinitrobenzenesulfonic acid (TNBS) assay.

### Preparation of nanoparticles

Nanocomplexes were prepared in HBG buffer (HEPES buffered glucose solution; 20 mM HEPES, 5% glucose, pH = 7.2). Branched PEI derivatives were prepared at different concentrations and 50 ul of the solution containing bPEI was added to the same volume of plasmid DNA solution (40 μg/ml). The mixture was incubated for 20–30 min at room temperature in order to allow the formation of stable polyplexes. The composition of the prepared formulations was defined by C/P ratio in which C is the weight of bPEI and its derivatives and P represents the weight of plasmid DNA used for the complex formation.

### Evaluation of buffering capacity

A simple acid-base titration method was used to measure the buffering capacity of PEI and its derivative as described elsewhere^9,10^. Buffering capacity, β, was calculated using the following equation:

$${\rm{Buffer}}\,{\rm{capacity}}\,({\rm{\beta }})={{\rm{\Delta }}\text{Acid}}_{{\rm{mol}}}/{\rm{\Delta }}\text{pH}$$

### Evaluation of plasmid DNA binding affinity

In order to evaluate the binding strength of plasmid DNA to the unmodified bPEI and its conjugate, ethidium bromide (EtBr) exclusion assay was carried out. The exclusion of the EtBr from plasmids following the addition of bPEI and its derivative leads to a decrease in fluorescence intensity. The decrease of fluorescence intensity is an indicator to show the ability of polycationic compounds to condense plasmid DNA. The fluorescence intensity of a solution containing plasmid DNA (400 ng/ml) and EtBr (0.4 mg/ml) in HBG buffer was set to 100% using a spectrofluorometer (LS55, PerkinElmer, MA, USA). Following the addition of bPEI or its conjugate to the plasmid solution, the fluorescence intensity was recorded. The polymer solution was added stepwise to the plasmid DNA solution and the decrease of fluorescence intensity was recorded (excitation wavelength = 510 nm and emission wavelength = 590 nm). The quantitative binding affinity of bPEI and its derivative to plasmid DNA was shown by plotting the relative fluorescence intensity versus C/P ratios.

### Particle size and ζ potential measurement

The particle sizes and ζ potential of polyplexes were determined using Dynamic Light Scattering (DLS) and Laser Doppler Velocimetry (LDV), respectively, using Malvern Nano ZS (Malvern Instruments, Malvern, UK). The polyplexes were prepared in HBG buffer at C/P ratio of 8 by mixing the equal volumes of the buffer containing bPEI and plasmid DNA. Data were collected for 30 cycles in automatic mode and the results were reported as mean ± SD (n = 3).

### DNase I protection assay

In order to evaluate the ability of bPEI and its conjugate in the protection of plasmid DNA against enzymatic digestion, DNase I protection assay was carried out as described elsewhere^27^. Briefly, the polymer/plasmid DNA complexes were prepared at various C/P ratios (i.e; C/P ratios of 0.25, 4 and 8) and mixed with 1 µL of DNase I and incubated for 30 min at 37 °C. Then, EDTA (250 mM) was added for enzyme deactivation followed by the addition of sodium dodecyl sulphate (1%). After 2 hours of incubation at room temperature, the complexes were run on agarose gel to evaluate the protection ability of polycationic compounds.

### Atomic force microscopy (AFM) and scanning electron microscopy (SEM)

Atomic force microscopy (JPK NanoWizard IIinstrument, Berlin, Germany) and scanning electron microscopy (VEGA TESCAN instrument, Brono,Czech Republic) were utilized to evaluate the morphological properties of the polyplexes. The statistical analysis of particles height from AFM images calculated by Gwyddion software for more than 200 particles. The complexes were dispersed on the copper tapes and then coated with gold in the ionization chamber for SEM analysis.

### Cell culture and transfection procedure

The human ovarian cancer cell line, OVCAR-3 (C430, NCBI, Tehran, Iran) and HepG2 hepatocellular carcinoma cell (C158, NCBI, Tehran, Iran) were maintained at 37 °C, 5% CO_2_ and 100% humidity in RPMI 1640 medium supplemented with 10% fetal bovine serum (FBS), streptomycin at 100 μg/ml and penicillin at 100 IU/ml. *In vitro* transfection experiments were carried out using IL-12 plasmid (pUMVC3-hIL12) at the final concentration of 0.2 μg plasmids/well. One day before starting the transfection experiments, the cells were seeded at a density of 1 × 10^4^ cells/well in 96-well plates. Various formulations of polyplexes at different C/P ratios (i.e; 0.25, 4 and 8) were prepared as described earlier. Then, 10 μl of each formulation was added to each well and incubated for 4 h at 37 °C followed by the medium replacement and incubation for 48 h in order to allow the expression of the transgene.

### Specificity of transfection

Competitive inhibition of the integrin receptors was evaluated by L-thyroxine as the free targeting ligand of α_v_β_3_ integrin receptor. OVCAR-3 cells were seeded as described above and pre-treated with L-thyroxine at different concentrations (i.e.; 10^−10^ to 10^−3^ mol/ml) for 30 min at 37 °C. In order to prevent the conversion of L-thyroxine (T_4_) to 3,5,3′-triiodothyronine (T_3_), propylthiouracil (1 mM) was used to inhibit tetraiodothyronine 5′ deiodinase. Then, the different formulation of complexes were prepared and added to each well for 4 h. The expression of hIL-12 was evaluated 48 h later.

### Evaluation of the expression of hIL-12

The expression of hIL-12 gene was measured using the human IL-12 (p70) ELISA kit (BD Bioscience, Heidelberg, Germany), according to the manufacturer’s instructions. Briefly, following the coating of ELISA plates by capture antibody and addition of assay diluent to the wells, controls, standard and samples were added. Then, the detection monoclonal antibody and the substrate solution were added to each well. Following the addition of H_3_PO_4_ (1 M), the absorbance was recorded with an ELISA plate reader at 450 nm.

### Cell viability assay

The viability test was carried out at the same condition used in the transfection experiments. The MTT assay was performed by the addition of 10 μl of complex formulations to each well and incubated for 4 h. Then, the replacement of the medium was carried out and the incubation continued for 48 h. Finally, the medium was aspirated and MTT solution (5 mg/ml) was added to the wells and incubated for around 1.5 h at 37 °C. Following the addition of dimethyl sulfoxide (DMSO) to the wells, the formazan crystals were dissolved and the absorbance was measured by an ELISA reader (ELx800, BioTek, Germany) at 590 nm and background corrected at 630 nm. The cell viability (%) relative to control wells not treated with nanoparticles was calculated by [A] test/[A] control × 100.

### Radiolabeling with ^99m^Tc

bPEI and its conjugate were labeled with ^99m^TcO_4_^−^ by direct labeling method as described elsewhere^28,29^. Briefly, 100 μl of ^99m^TcO_4_^−^ (2 mCi) in normal saline (0.9% w/v) was added to 100 μl (1 mg/ml) of bPEI solutions followed by the addition of 10 μl stannous chloride (2 mg/ml in 0.1 M HCl) and adjustment of pH to 6 by NaOH (0.1 M). Finally, the resulting solution was incubated at 37 °C for 30 minutes. The radiolabeled formulations were stored at sealed vials for further investigations. All the procedures for radiolabeling were approved by the nuclear medicine advisory committee of Shiraz School of Pharmacy (Shiraz, Iran).

### Imaging and biodistribution studies

All animal experiments were approved by institutional ethical committee and research advisory committee of the Shiraz University of Medical Sciences (Shiraz, Iran) based on the ethical guidelines for the care and use of animals in medical research. Female Balb/c mice were obtained from the animal laboratory of Shiraz University of Medical Sciences. Each mice received 5.55 MBq (150 μl) dose of the labeled polyplexes via the tail vein intravenous injection. The static images were obtained with a single-headed gamma camera (Nucline TH, Mediso, Hungary) equipped with a low energy general purpose (LEGP) collimator. Gamma camera images were acquired at 30 to 240 min after injection. Finally, the mice were sacrificed at 0.5, 1, 2, and 4 hours post injection and various organs including kidney, brain, heart, spleen, liver and intestine were dissected. The collected organs were washed with normal saline and dried followed by weighting and their activity was measured by dose calibrator. The biodistribution of radiolabeled polyplexes in each organ was measured as a percentage of the injected dose per gram of tissue. The difference between the original syringe activity and the remaining activity after the injection was considered as the total activity injected per animal. Correction was made for background radiation and physical decay during counting^28,29^.

### Statistical analysis

Student’s t-test was used to analyze the data. A probability value of less than 0.05 was considered significant. Results were expressed as mean ± standard deviation (SD).

### Data availability

All data generated or analyzed during this study are included in this published article (and its Supplementary Information files).

## Results and Discussion

### Synthesis of conjugated bPEIs

**Figure 1:**
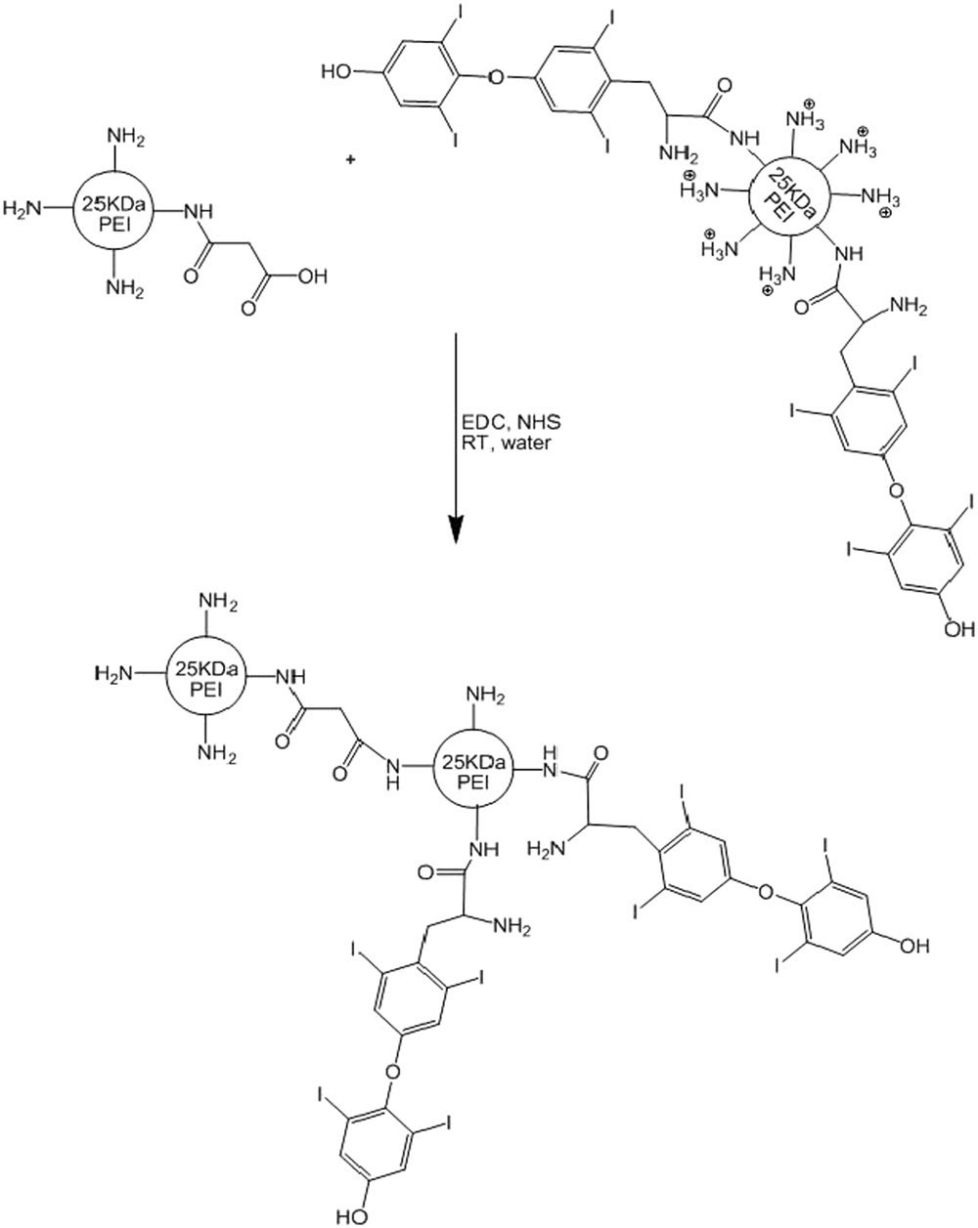
Synthesis of bPEI derivative. Branched PEI (25 kDa) was initially modified by succinic anhydride. Another bPEI molecule was conjugated with L-thyroxine. These two domains were coupled through an amide bond.

In order to prepare L-thyroxine conjugated bPEI coupled with the succinated bPEI, a three step synthesis strategy was utilized as shown in Fig. 1. First, the bPEI primary amines were conjugated by succinic anhydride to provide the terminal carboxylate group for the formation of the further amide bond. The succinated bPEI structure was confirmed by ^1^H-NMR and FT-IR as described previously^26^ and labeled as PEI-SUC conjugate. On the other hand, the conjugation of L-thyroxine to the amines of another bPEI molecule was carried out by 1-ethyl-3-[3-dimethylaminopropyl] carbodiimide hydrochloride (EDC) through the amide bond formation. The conjugation of L-thyroxine through the surface amines of bPEI was confirmed as described in our previous investigation^14^. The degree of L-thyroxine grafting was expressed as a number of conjugations per bPEI monomer unit ×100%. By adjusting the reagent/PEI amine ratio in the initial feed, the desired conjugation degree of 10% was achieved. The degree of L-thyroxine grafting was determined by ^1^H-NMR from the ratio between the peaks of bPEI (δ 2.7–3.2 ppm) and the aromatic rings of L-thyroxine (δ 7.2–7.9 ppm). The L-thyroxine conjugated bPEI was coded as PEI-LT. Finally, the double domain conjugate of bPEI was synthesized by the amide bond formation between the PEI-SUC and PEI-LT conjugates using 1-ethyl-3-[3-dimethylaminopropyl] carbodiimide hydrochloride (EDC) and N-hydroxybenzotriazole. According to ^1^H-NMR spectra, bPEI protons appeared between 2.7–3.2 ppm while the L-thyroxine protons were found between 7.00 and 7.69 ppm. According to FT-IR spectra, two peaks at 1658 and 1715 cm^−1^ were found for PEI SUC conjugate corresponding to amide and carboxylic acid groups, respectively. Following the conjugation of PEI-LT to the PEI-SUC structure, the carboxylate peak weakened to 1660 cm^−1^ confirming the formation of an amide bond. The final conjugate was labeled as PEI-SUC-PEI-LT.

## Biophysical characterization of polymer and polyplexes

### Buffering capacity assessment

**Figure 2:**
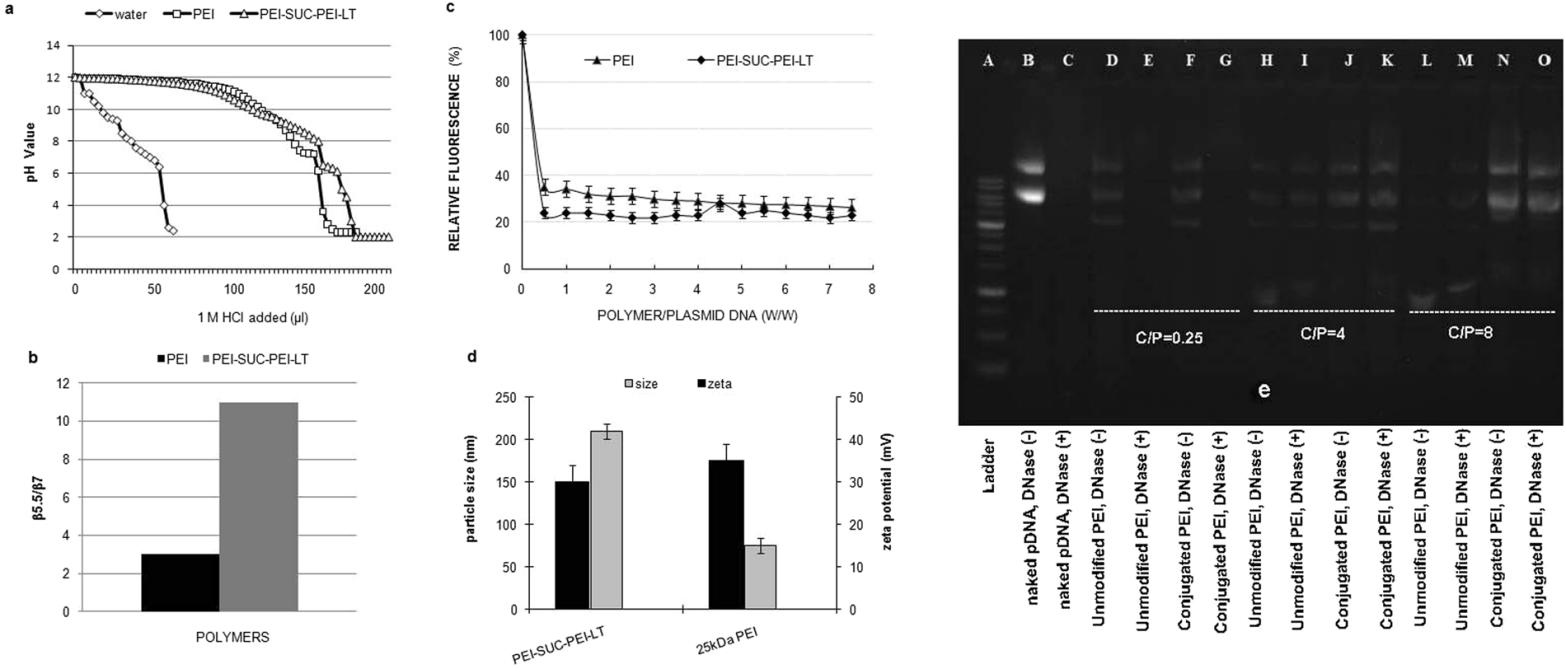
Biophysical characterization of polymer and polyplexes: (**a**) Buffering capacity measurement. Titration curves for unmodified bPEI and its derivative, PEI-SUC-PEI-LT, from pH 12 to 2. (**b**) The comparison between buffering capacity of unmodified bPEI and PEI-SUC-PEI-LT derivative. (**c**) Plasmid DNA binding affinity by bPEI and its derivative. (**d**) Particle size and ζ potential of the polyp lexes at C/P = 8. (**e**) DNase I protection assay. Cropping lines are used in the figure. Full-length gels are presented in Supplementary Fig. S1.

One of the major barriers limiting the efficient transfer of genetic materials into their final target inside the cells is endo/lysosomal compartments. These vesicles induce the degradation of nucleic acid materials by the acid hydrolases at low pH. It has been suggested that the high amine content of PEI acts as a proton sponge capturing the protons during the process of endosome acidification which results in the efflux of chloride ion and subsequently water into the compartments^30^. Finally, increased osmotic pressure leads to the vesicle swelling and disruption of endo/lysosome membrane which results in the early escape of nucleic acid materials into the cytosol before starting the degradation process. The critical pH range for this process is around 5.5–7^30^. The mechanism by which the PEI molecules induce the early escape of polyplexes from endosomes is called ‘proton sponge effect’. Generally, this mechanism has been widely accepted by researchers as the major mechanism of PEI gene delivery into the target cell efficiently^13^. In order to measure the buffering capacity of unmodified bPEI and its conjugate, an acid-base titration method was carried out. The intrinsic capacity of the bPEI and its derivative to act as a buffering agent was shown by plotting the changes of pH *versus* the added acid. As demonstrated in Fig. 2a, unmodified 25 kDa bPEI demonstrated remarkable buffering capacity over almost the entire pH range whereas the PEI-SUC-PEI-LT conjugate with substantial amine content increased the buffering capacity in comparison with unmodified bPEI. According to the previous investigations, attachment of pendant moieties through the surface amines of bPEI may decrease the overall buffering capacity which leads to the lower transfection efficiencies^15,18,31^. However, low conjugation degrees and also the conversion of primary amines to the secondary ones with the pKa values around 9 might prevent decreased gene transfer ability^9,32^. According to the results of our previous study, the conjugation degree of L-thyroxine on the bPEI molecule was adjusted to around 10% which had been proved to be appropriate for significant buffering capacity^14^. The substantial buffering capacity of bPEI and its derivative in the pH range of 9–11 is associated with the primary and secondary amines whereas the tertiary amines with the reported pKa values around 6–7 might be responsible to induce considerable buffering capacity at the pH range of 5.5–7^33,34^. This is the crucial pH range which acts as the deriving force to induce the early escape of polyplexes from endosomal compartments^35^. As shown in Fig. 2b, the buffering capacity of the PEI derivative is considerably higher than the unmodified PEI in the crucial pH range of 5.5–7. In the present study, conjugation of two bPEI molecules resulted in the increase of total buffering capacity due to the increased amine content of the whole molecule. Despite some controversial investigations reporting the negligible impact of PEI buffering capacity in its high transfection efficiency^36^, the proton sponge effect is still the most widely accepted mechanism for the transfer of genetic materials into the cells by PEI-based nanocarriers.

### Binding strength of polymer to plasmid DNA

One major parameter determining successful gene delivery by polycationic compounds is the ability of these structures in condensation of oligonucleotides into compact nanostructures^11,37,38^. The formation of polymer/plasmid DNA complexes (i.e; polyplexes) results from the interaction between the plasmid phosphate backbone with negative charge and the amines of polymers with positive charge^38^. The binding strength of bPEI to the plasmid DNA could be measured using the ethidium bromide (EtBr) exclusion assay. Following the addition of polycationic compounds to the plasmid DNA solution, the intercalated EtBr excludes from the plasmid and leads to a decrease in the fluorescence intensity. Hence, more binding affinity of bPEI to plasmid DNA results in the more decrease of fluorescence intensity. The results of the fluorescence quenching measurement indicated that unmodified bPEI and its derivative were able to optimally condense plasmid DNA at carrier/plasmid ratios higher than 1 (Fig. 2c). As illustrated in the Fig. 2c, the pattern of fluorescence decrease following the addition of bPEI and its conjugate was similar. According to our previous results on the conjugation of succinic acid or L-thyroxine on the bPEI structure, those conjugates were also kept their binding affinity at the conjugation degrees less than 10%. In other word, the substitution of 10 mole% of the bPEI amines had no significant effect on its binding affinity. This means that the remaining positive charge on the polymer is still enough for efficient electrostatic interaction to form polyplexes. Since the amine content of PEI-SUC-PEI-LT conjugate was higher than the unmodified bPEI, it could be concluded that the condensation ability of the conjugate might be even higher than the parent polymer (Fig. 2c). At C/P = 0.25, the unmodified bPEI decreased the fluorescence intensity by around 35%, while the reduction of fluorescence intensity for PEI-SUC-PEI-LT derivative was approximately 24%. By the addition of more bPEI or its conjugate to the plasmid solution the fluorescence intensity reached to plateau state. There are several reports indicating that the conjugation of various moieties via the amines of PEI leads to the formation of complexes with lower binding affinity to plasmid DNA. These looser polyplexes may result in the facilitated dissociation of plasmid DNA from the carrier and consequently higher transfection efficiency^31,32,39^. However, the results obtained in this study and the results of our previous investigation on the conjugation of L-thyroxine onto bPEI structure^14^ revealed that the polymers with the highest binding affinity demonstrated the highest efficiency for plasmid DNA delivery. In the present study, the separation of targeting domain from the delivery domain might be resulted in the formation of tight complexes rather than the complexes formed by PEI-SUC or PEI-LT conjugates alone. The impact of plasmid DNA binding affinity on gene delivery is controversial. Totally, it seems that the dissociation of plasmid DNA from the polymer may not increase the gene transfer ability unless it is considered as the rate limiting step in transfection^34^. It has been shown that at the low carrier to plasmid ratios polyplexes are not stable and undergo aggregation resulting from the lack of sufficient positive charge on the surface of complexes to prevent their further aggregation. Higher carrier to plasmid ratios stabilizes the complexes in a sphere-like condensed structure which contains one DNA chain per polyplex. The surface of such structures contains a layer of anchored cationic branched PEI chains or small cationic bPEI tails and loops. Each branched chain of PEI not only neutralizes the negative charge of DNA but also binds various intrachain segments with its different sides to cross-link these parts together to form the complexes. This specific surface structure is able to stabilize each polyplex in the medium facilitating the complexes to be internalized through receptor mediated endocytosis^40^.

### Measurement of particle size and ζ potential

The size and surface charge properties of the polyplexes formed by the electrostatic interaction between the plasmid DNA and PEI play a crucial role in different steps of nucleic acid delivery by polycationic compounds including the mechanism by which the polyplexes enter the cells as well as cytotoxicity, protection against nucleases and their biodistribution. Having demonstrated that the PEI-SUC-PEI-LT conjugate was able to condense plasmid DNA and form the polyplexes, Dynamic Light Scattering (DLS) and Laser Doppler Velocimetry (LDV) were used to determine the particle size and ζ potential of the polyplexes formed at the optimum C/P ratio of 8 (*vide infra*). According to the results of particle size measurements (Fig. 2d), the unmodified bPEI formed the complexes with the size of around 75 nm whereas the PEI-SUC-PEI-LT conjugate formed polyplexes with the size of 210 nm. The larger particle size might be associated with the larger molecular size of the polymer used for the formation of complexes^41,42^. The results of LDV demonstrated that the polyplex ζ potential remained around 30 mV and no significant changes between the ζ potential of the unmodified polymer and its derivative has been observed. According to our previous results, the ζ potential of PEI-LT conjugate was around 17 mV^14^. The increase of charge density of the bPEI derivative prepared in the present investigation could be attributed with the attachment of positively charged bPEI molecule on the PEI-LT conjugate which provided higher positive charge density on the surface of the final derivative. This result was consistent with the binding affinity observations demonstrated the high strength of the PEI conjugate to interact electrostatically with the negatively charged plasmid DNA molecules. The particle size measurement was also carried out 2 h after the preparation of the complexes which showed the stability of the complexes during the incubation period. This might be associated with the sufficient positive charge on the polymer surface preventing the aggregation due to the charge repulsion between the polyplexes with positive charge.

### Protection of plasmid DNA against enzymatic digestion

Considering the high susceptibility of naked plasmid DNA to various enzymes including nucleases, condensation of nucleic acids by polycationic compounds not only forms the comlexes with favorable size and charge properties but also protects the plasmid DNA to be degraded by digesting enzymes. In order to assess the ability of bPEI and its derivative to protect plasmid DNA against enzymatic degradation, DNase I was used as the model enzyme and the protection effect of the polymers on plasmid DNA was demonstrated by agarose gel electrophoresis. As illustrated in Fig. 2e, neither unmodified bPEI nor its derivative could protect plasmid DNA at C/P = 0.25 whereas the significant protection effect was observed at the higher C/P ratios. Interestingly, the protection effect of the bPEI derivative was relatively higher than the unmodified bPEI. At the same condition, naked plasmid DNA was completely digested by the enzyme. Since the charge density and binding affinity of the complexes formed by bPEI conjugate was higher than those formed by the unmodified polymer, it could be concluded that these properties resulted in the more protection against nuclease digestion^42^. However, there are some reports indicating that more binding affinity or charge density will not necessarily lead to more protection against enzymatic degradation^40,43^. It seems that there are some other polyplex characteristics determining the protection effect of polycations on plasmid DNA such as their shape and conformation. Since the polyplexes form following the interaction between the polymer and plasmid by electrostatic interactions, some plasmid segments may be oriented towards the exterior of the complexes. These segments show more susceptibility to be digested by enzymes. On the other hand, high amine content of the polycations may lead to the formation of polyplexes by “wrap around” the plasmid DNA resulting in more protection effect^14,40,43^.

### AFM and SEM analyses

**Figure 3:**
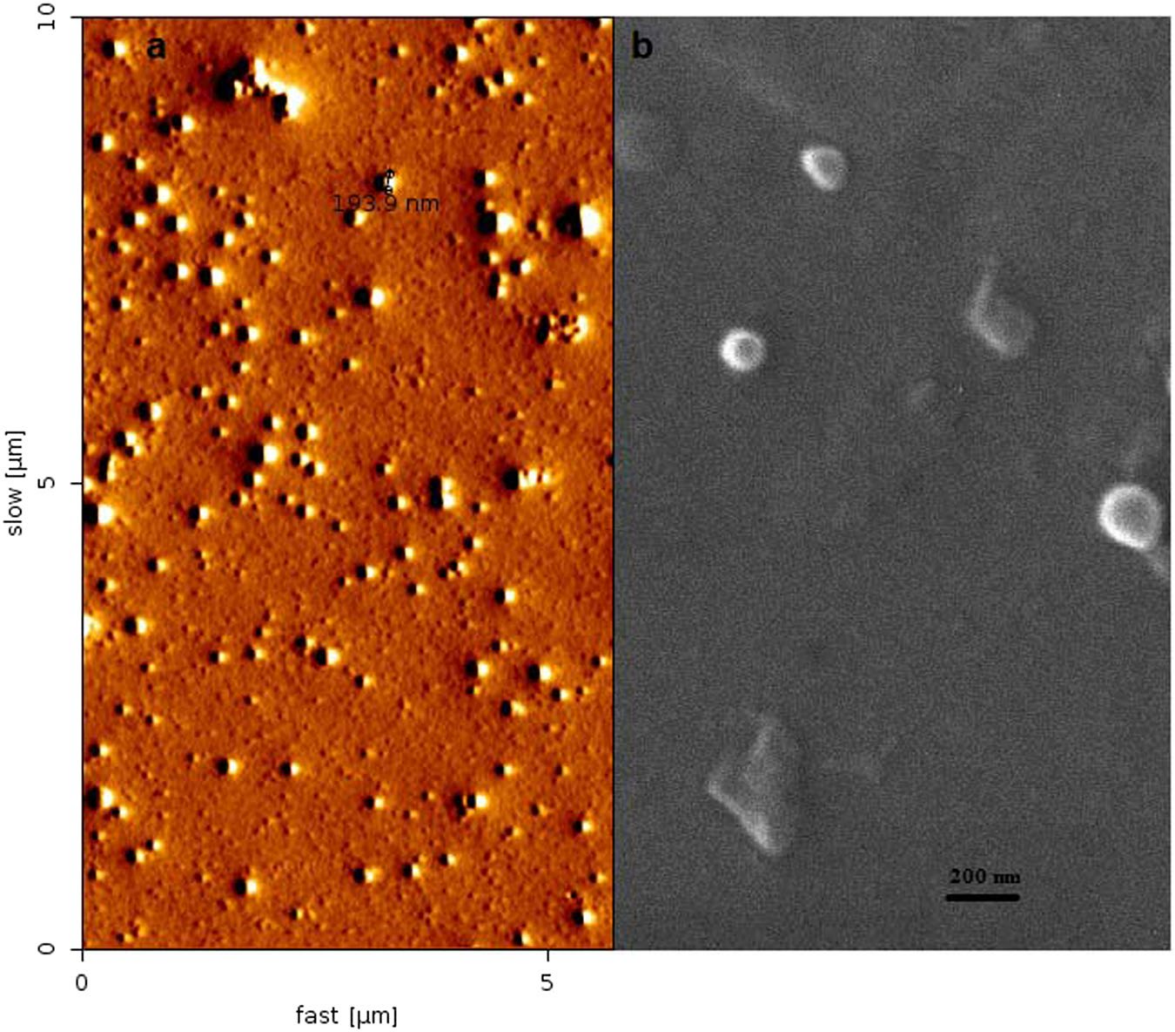
(**a**) Atomic force microscopy and (**b**) scanning electron microscopy micrographs of the polyplexes at C/P ratio of 8. The AFM image with 11 different line profiles to calculate the height profile of particles and the histogram are presented in Supplementary Figs S3–S5. Cropping lines are used in the figure. Full-length micrographs are presented in Supplementary Figs S2 and S6.

AFM and SEM were used for further morphorogical characterization of the polyplexes at the C/P ratio of 8 (Fig. 3a,b). The size of the complexes was relatively similar to the DLS analysis. (Fig. 2d). However, the limitations and specifications of these techniques including the observation of a small fraction of complexes and evaluation of individual complexes must be considered. The AFM image with 11 different line profiles was used to calculate the height profile of the particles (See Figs S3 and S4). AFM analysis indicated the particles histogram with height of 16.9 ± 9.4 nm (see Fig. S5).

### Transgene expression

**Figure 4:**
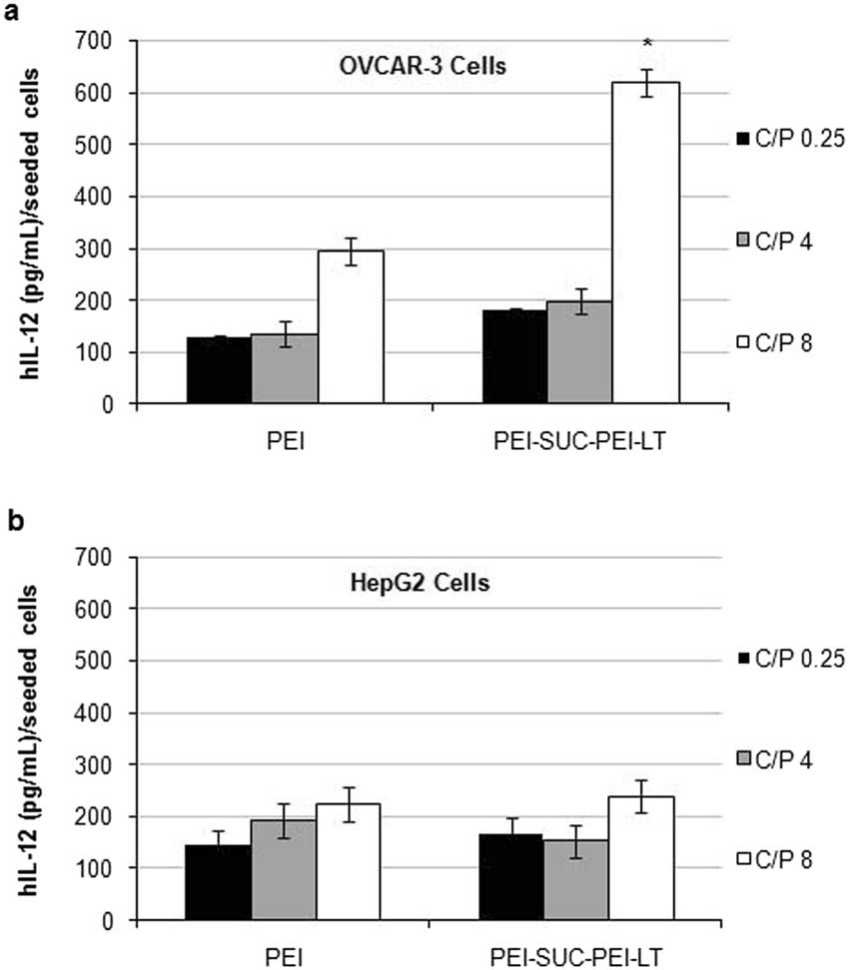
Gene transfer ability of bPEI and its conjugate. (**a**) The levels of hIL-12 in OVCAR-3 cells and (**b**) HepG2 cell following the treatment with polyplexes at C/P ratios of 0.25, 4 and 8. The level of hIL-12 expression was presented as the concentration of the protein (pg/ml) per seeded cells. *p < 0.05, conjugated bPEI derivative compared to unmodified parent polymer at the same C/P ratio (N = 3; error bars represent ± standard deviation).

Recombinant protein of IL-12 has been considered as a potential anti tumor agent through cytokine mediated immunotherapy. However, its wide clinical application has been limited due to the deaths reported following the systemic administration of the protein in the patients with cancer^2,5^. Therefore, researchers are seeking for the alternative strategies to utilize the protein while reducing its systemic adverse reactions. As an alternative route, the transfer of the gene encoding hIL-12 into the tumor site has been proposed. Based on this method, the production of the therapeutic protein occurs within the target tissue or organ and the elevated levels of IFN-γ will be limited to the tumor site. This may reduce the systemic adverse reactions associated with the increased levels of IFN-γ as the main mediator of lethal reactions^9,14,42,44–46^. In the present study, a double domain bPEI-based nanocarrier was prepared based on the separation of the targeting domain from the plasmid delivery domain and the gene transfer ability of the polyplexes prepared by the bPEI and its conjugate was evaluated in the OVCAR-3 cell line with high expression of integrin α_v_β_3_ and HepG2 cells with very low levels of the receptor. As demonstrated in Fig. 4a,b, the level of the transgene expression using the unmodified bPEI was in the range of 150–300 (pg/ml)/ seeded cells in the both cell lines and there was no difference between the transfection efficiency of non-targeted polyplexes at any C/P ratio in the cell line over-expressing the integrin α_v_β_3_ and the cell line with low levels of the receptor (*p* > 0.05). On the other hand, PEI-SUC-PEI-LT derivative increased the level of hIL-12 by up to two folds compared with unmodified bPEI at the same C/P ratio. The highest expression level occurred at C/P = 8 in which the highest protection effect was also observed (Fig. 3a). The degree of L-thyroxine conjugation on the bPEI surface was optimized to around 10 mole% of the primary amines of bPEI based on our previous study^14^. According to our investigations, the highest level of integrin receptor mediated delivery by the PEI polymers occurred at the conjugation degrees between 5–10%. The conjugation degree of the targeting moieties plays a crucial role in the targeted delivery of carriers to the cells. Once the lower substitution degree is not able to substantially increase the transfection, the higher degrees of grafting may also reduce the gene transfer ability of the polymers. Higher conjugation degrees may lead to strong extracellular adherence which consequently results in the reduced internalization into the target cells^47^. Generally, the entrance of non targeted PEI-based nanoparticles occurs through adsorptive endocytosis which is the result of electrostatic interaction between the polyplexes with positive charges and the components on the cell membrane with negative charges. positively charged polyplexes and the negatively charged components on the cell membrane (e.g. sulfated proteoglycans)^12,13,30^. Although some nanoparticles penetrate the cell membrane directly, the PEI based polyplexes are supposed to enter the cells via non-specific adsorptive endocytosis^48^. Also there are some reports indicating the importance of such electrostatic interactions for further internalization through endocytosis^48,49^. On the other hand, incorporating targeting ligands to the polycations structure has been utilized to combine the non-specific electrostatic polyplex–cell surface interaction with the specific mechanism of receptor-mediated cellular uptake. The concept of receptor mediated gene delivery is based on the mechanisms widely used by toxins and viruses which employ natural cellular internalization processes such as receptor-mediated endocytosis, potocytosis, and phagocytosis. The unmodified non-targeted polymers enter the cells through an adsorptive endocytosis mechanism^48^ while the entrance of the L-thyroxine conjugated polymers is supposed to be by a zippering mechanism which enables the integrin-mediated uptake of large species including bacteria^9,14,49^.

### Specificity of gene delivery

**Figure 5:**
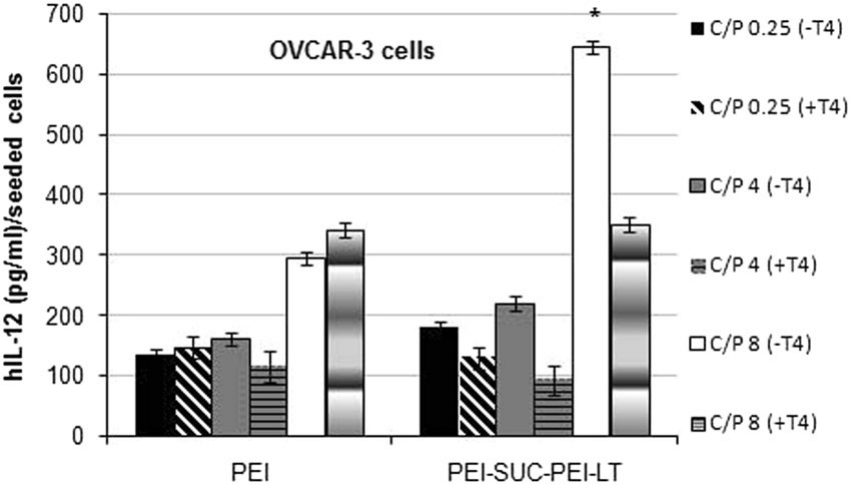
Specificity of transfection of bPEI and its derivatives complexed with pUMVC3-hIL12 plasmid in OVCAR-3 cell lines. L-thyroxine was used at the concentration of 10^−3^ mol/ml for competitive inhibition of the receptors. *p < 0.05, PEI-SUC-PEI-LT in the absence of L-thyroxine compared to the same derivative in the presence of L-thyroxine. (N = 3; error bars represent ± standard deviation).

The impact of α_v_β_3_ integrin receptors on the enhanced delivery of the plasmid DNA was demonstrated by the competitive inhibition of integrin receptors using L-throxine (T_4_) at the final concentrations of 10^−10^, 10^−7^ and 10^−3^ mole/ml. The results demonstrated that there was no significant difference between the gene transfer ability of bPEI derivative and its parent unmodified form at the same C/P ratios (Fig. 5). In other word, pre-treatment of the integrin over-expressing cells using L-thyroxine resulted in a substantial decrease in gene delivery. This test was also performed at the concentrations of 10^−10^ and 10^−7^ mole/ml which led to similar decrease in gene transfer ability of targeted PEI derivative. Hence, it could be concluded that integrin receptors play a major role in the entrance of PEI conjugate to OVCAR-3 cells.

### Cell viability assay

**Figure 6:**
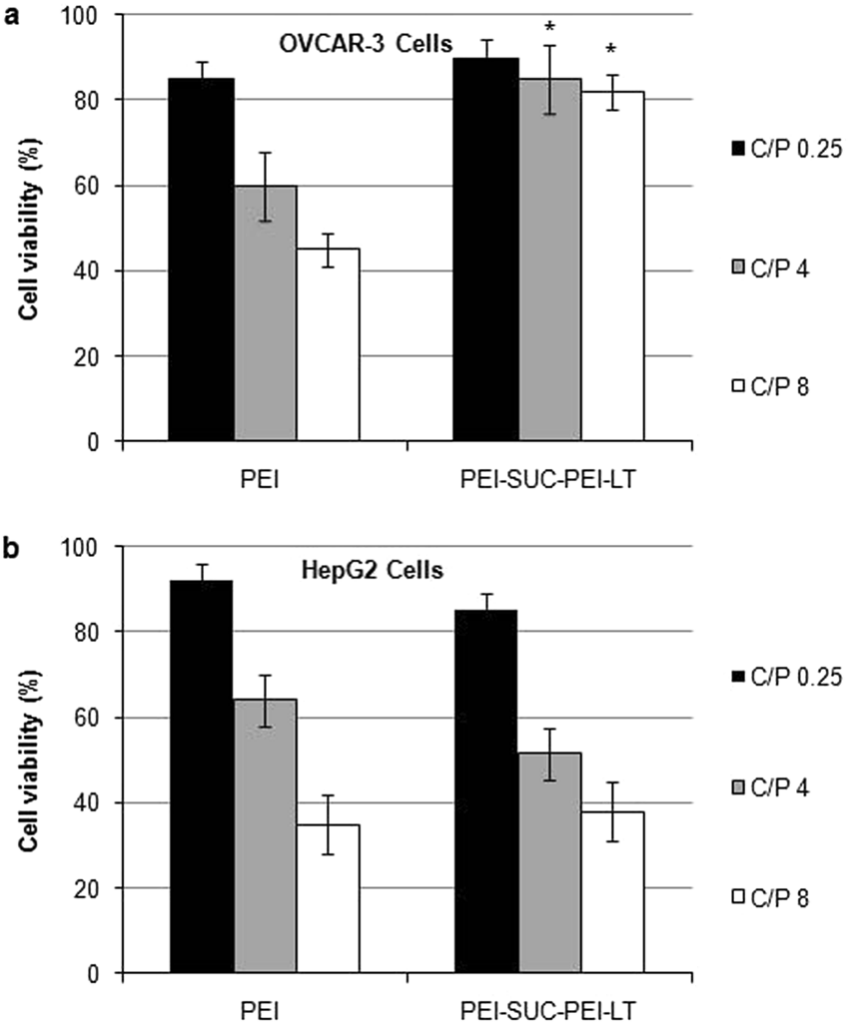
Viability of bPEI and its conjugate complexed with pUMVC3-hIL12 plasmid at C/P ratios of 0.25, 4 and 8 evaluated by MTT assay in (**a**) OVCAR-3 and (**b**) HepG2 cell lines. *p < 0.05, PEI-SUC-PEI-LT compared to unmodified PEI at the same C/P ratio. (N = 3; error bars represent ± standard deviation).

The viability of unmodified bPEI and its derivative was evaluated in OVCAR-3 and HepG2 cell lines by MTT assay at the same condition used in transfection experiments (Fig. 6). The results of cell viability experiments revealed a dose-dependent cytotoxicity on HepG2 cell line. The unmodified bPEI nanoparticles led to the cell viability around 40% while the cells treated by the polyplexes at C/P = 8. On the other hand, the viability of the cells following the treatment by polyplexes at C/P = 0.25 was around 85%. In other words, the amount of the polymer is the determining factor in the induction of toxicity in HepG2 cells. In HepG2 cells, no significant difference in cell viability between the unmodified bPEI and its derivative at the same C/P ratio was observed (*p* > 0.05). In OVCAR-3 cell line, the unmodified bPEI resulted in the highest toxicity at C/P = 8; whereas the cell viability was around 85% at the lowest C/P of 0.25. In other words, the toxic effects of unmodified bPEI increased by increasing the amount of polymer used for the preparation of polyplex. Interestingly, the PEI-SUC-PEI-LT derivative resulted in a substantially less toxicity in OVCAR-3 cells at all the C/P. According to our results, the cell viability of the bPEI conjugate in OVCAR-3 was around 80% even at the C/P = 8. The toxic effects of bPEI and its conjugate at higher C/P ratios in HepG2 cell line could be associated with the high positive charge density of the polyplexes. It has been shown that the positive charges on the polyplex surface interact with the components on the cell membrane with negative charge which results in the membrane disruption and cell death. The main factor inducing cell death following the treatment of the cells by polycationic compounds is their high positive charge density. However, unmodified bPEI resulted in toxic effects on the OVCAR-3 cells while the toxic effects of its conjugate remarkably decreased despite the increased surface positive charge at the same condition. For instance, the cell viability following the treatment of OVCAR-3 cells with unmodified PEI was around 40% which increased to 80% in the case of PEI-SUC-PEI-LT at the C/P = 8. The reduction of toxic effects of PEI-SUC-PEI-LT conjugate might be associated by the fact that the receptor-mediated endocytosis of the polyplexes reduces the concentration of the positively charged complexes in the medium which in turn lead to lower toxicity on the cells. Our results were consistent with the other investigations reporting the lower toxicity of bPEI and its derivatives following the attachment of ligands for receptor mediated delivery^9,50^. Since the non-targeted PEI derivatives enter the cells through adsorptive endocytosis, their accumulation in the medium lead to higher toxic effects^9,15,18,50,51^.

### Imaging and biodistribution studies

**Figure 7:**
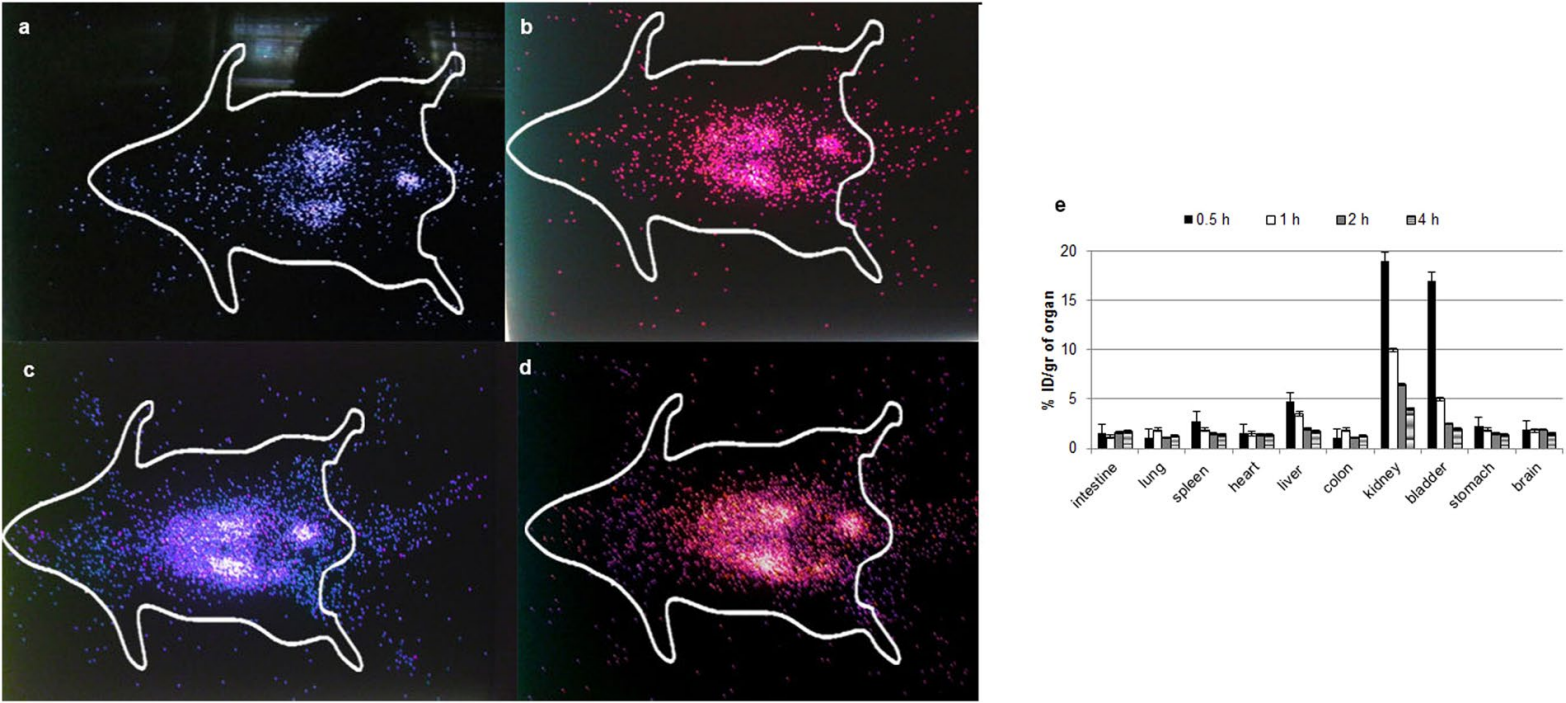
(**a**–**d**) Gamma images of mice treated with ^99m^Tc-bPEI/plasmid DNA complexes 0.5, 1, 2 and 4 h post injection. Original images are presented in Supplementary Figs S7–S10. (**e**) The scintigraphic analysis.

The investigations on the biodistribution of the radiolabeled polyplexes and imaging studies were carried out in Balb/c mice. In order to radiolabel the polyplexes, ^99m^Tc was utilized due to its low radiation dose, short half life and affordable cost as well as easy availability. Following the injection of ^99m^Tc-labeled bPEI/plasmid DNA complexes in to the mice, the polyplexes accumulated in kidney, bladder and liver during the first 0.5 h post injection (Fig. 7). After 4 h, the percentage of injected dose (ID) per gram of an organ raised from 4% to 18% and from 2% to 15% in the case of kidney and bladder, respectively. The maximum radioactivity found in kidney and bladder indicated the excretion of the radiolabeled polyplexes through kidney. Also, it has been shown that a small part of the polyplexes was taken up by liver and spleen after 4 h. The high accumulation of the polyplexes in kidney might be resulted from the specific characteristics of bPEI conjugate such as high hydrophilicity and water solubility^29^. The accumulation of the polyplexes in liver was significantly lower than the kidney indicating that the high hydrophilicity of the polyplexes led to bypassing the first pass effect of the organs such as liver^52^. Since these nanoparticles accumulated in kidney and bladder, the tumors in these organs may be a good choice for further studies. Also, increasing their retention time in blood circulation by different approaches (e.g; PEGylation) may be considered for next investigations. Since the nanoparticles did not show first-pass effect, theses delivery systems might be suitable for delivery of drugs showing high first pass effect and degradation in liver.

## Conclusion

Using simple conjugation methods, succinated bPEI and the L-thyroxine conjugated bPEI were coupled through an amide bond. The results revealed that preparation of the L-thyroxine PEI conjugate might be an effective strategy to transfer genetic materials into the target cells while maintaining the high binding affinity and buffering capacity of the PEI molecule.

## Electronic supplementary material

Supplementary Information
