# Supplementary material for: Double domain polyethylenimine-based nanoparticles for integrin receptor mediated delivery of plasmid DNA
Source: Sci Rep. 2018 May 1;8:6842. doi: 10.1038/s41598-018-25277-z (PMC5931586; doi:10.1038/s41598-018-25277-z)
Supplement: Supplementary file 1 — Supplementary Information [file 41598_2018_25277_MOESM1_ESM.doc]

**Double domain polyethylenimine-based nanoparticles for integrin receptor mediated delivery of plasmid DNA**

**Hossein Sadeghpour^1,2^, Bahman Khalvati^3,4^, Elaheh Entezar-Almahdi^1,4^, Narjes Savadi^1,2^, Samira Hossaini Alhashemi^1^ , Mohammad Raoufi ^5^& Ali Dehshahri^1,4,*^**

^1^Pharmaceutical Sciences Research Center, Shiraz University of Medical Sciences, Shiraz, Iran.

^2^Department of Medicinal Chemistry, School of Pharmacy, Shiraz University of Medical Sciences, Shiraz, Iran.

**^3^Medicinal Plants Research Center, Yasuj University of Medical Sciences, Yasuj, Iran.**

^4^Department of Pharmaceutical Biotechnology, School of Pharmacy, Shiraz University of Medical Sciences, Shiraz, Iran.

^5^Department of Nanotechnology and Nanotechnology Research Center, Faculty of Pharmacy, Tehran University of Medical Sciences, Tehran, Iran.

*Correspondence and requests for materials should be addressed to A.D. (email:dehshahria@sums.ac.ir)

Supplementary Fig.S1. Full length gel result of Fig. 2e.


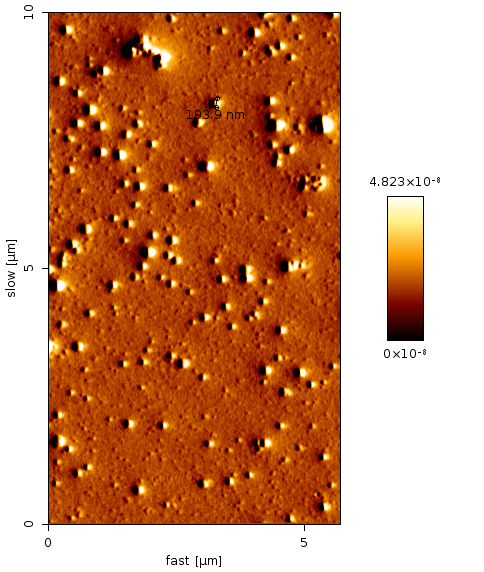


Supplementary Fig.S2. AFM micrographs of Fig. 3a.


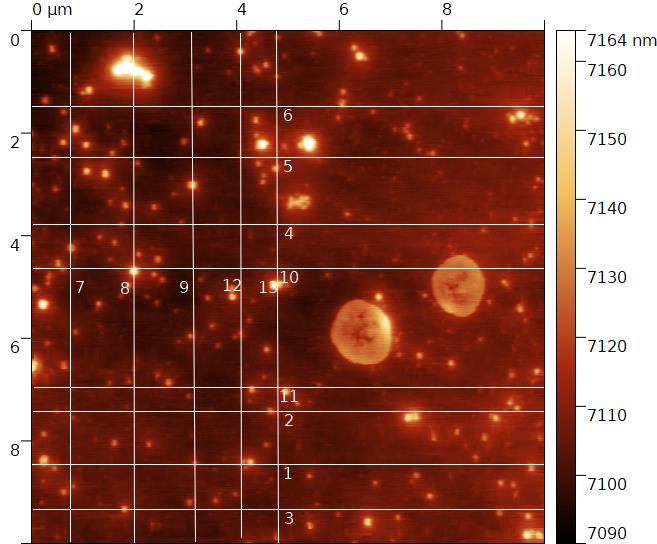


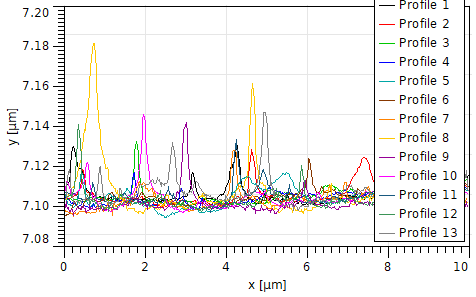


Supplementary Fig.S3 and S4. The AFM image with 11 different line profiles to calculate the height profile of particles.


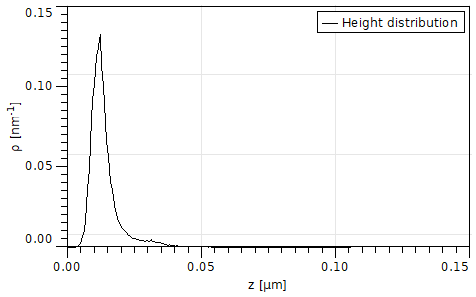


Supplementary Fig.S5. The particles histogram of AFM image.


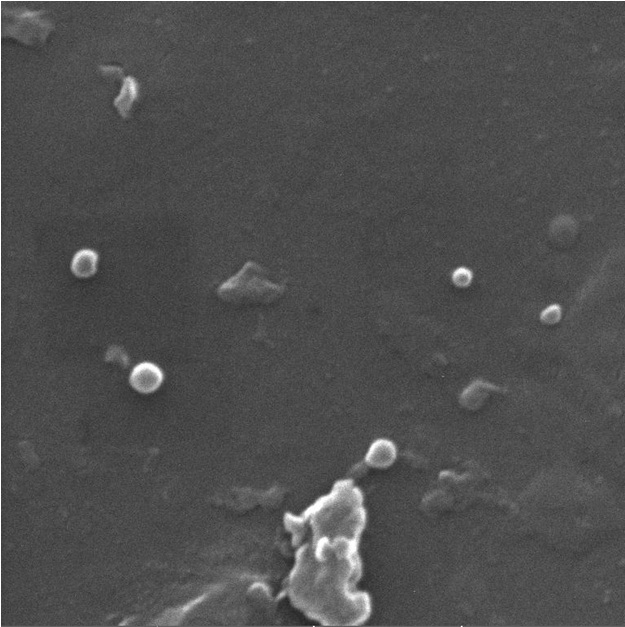


Supplementary Fig.S6. SEM micrographs of Fig.3b.


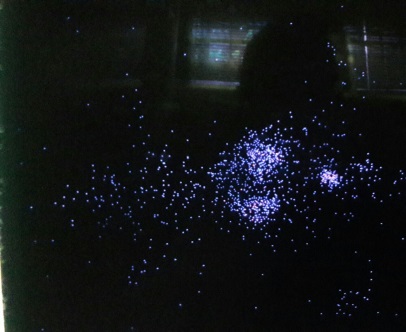


Supplementary Fig.S7. Gamma images of mice treated with ^99m^Tc-PEI/DNA complexes 0.5 h post injection.


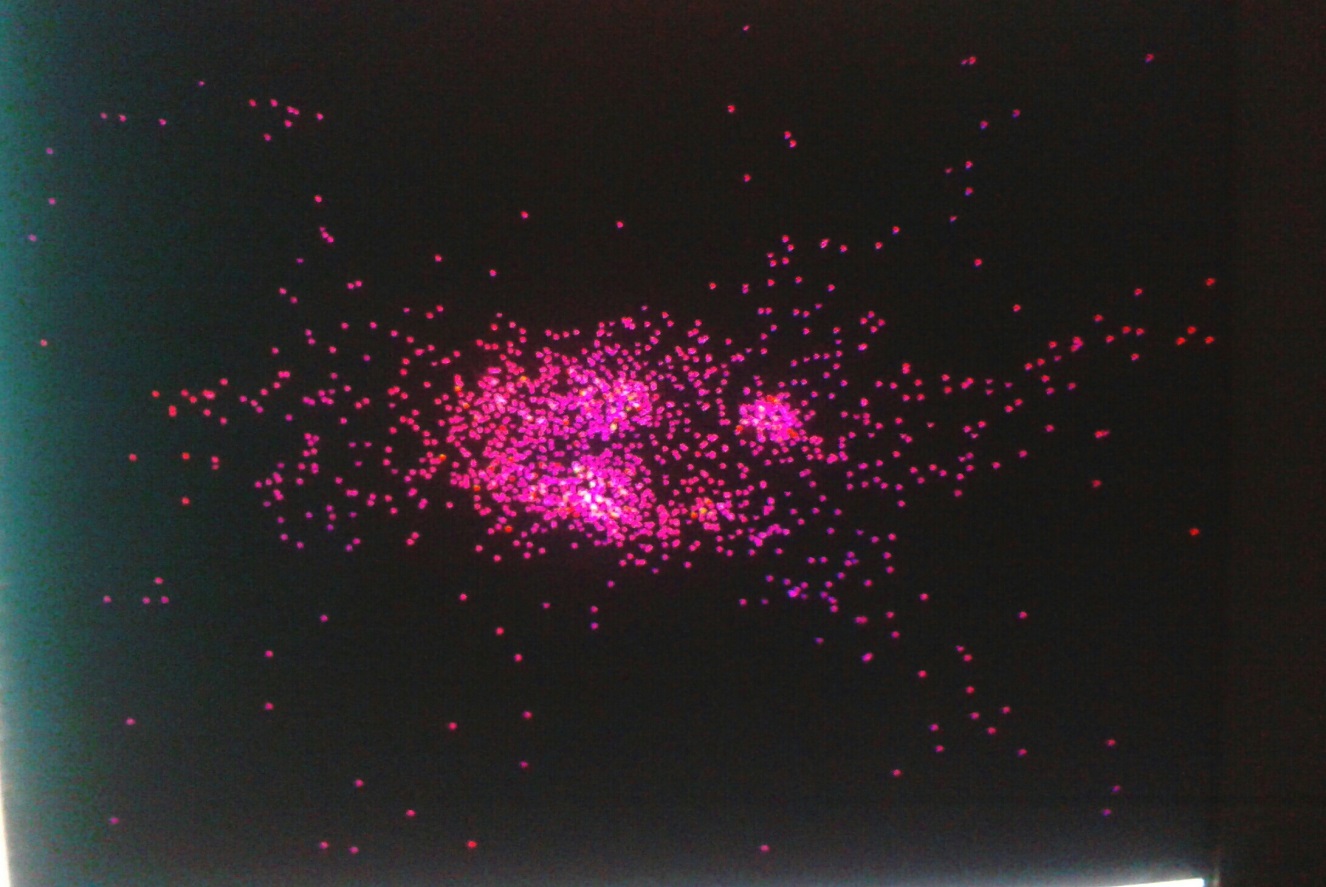


Supplementary Fig.S8. Gamma images of mice treated with ^99m^Tc-PEI/DNA complexes 1 h post injection.


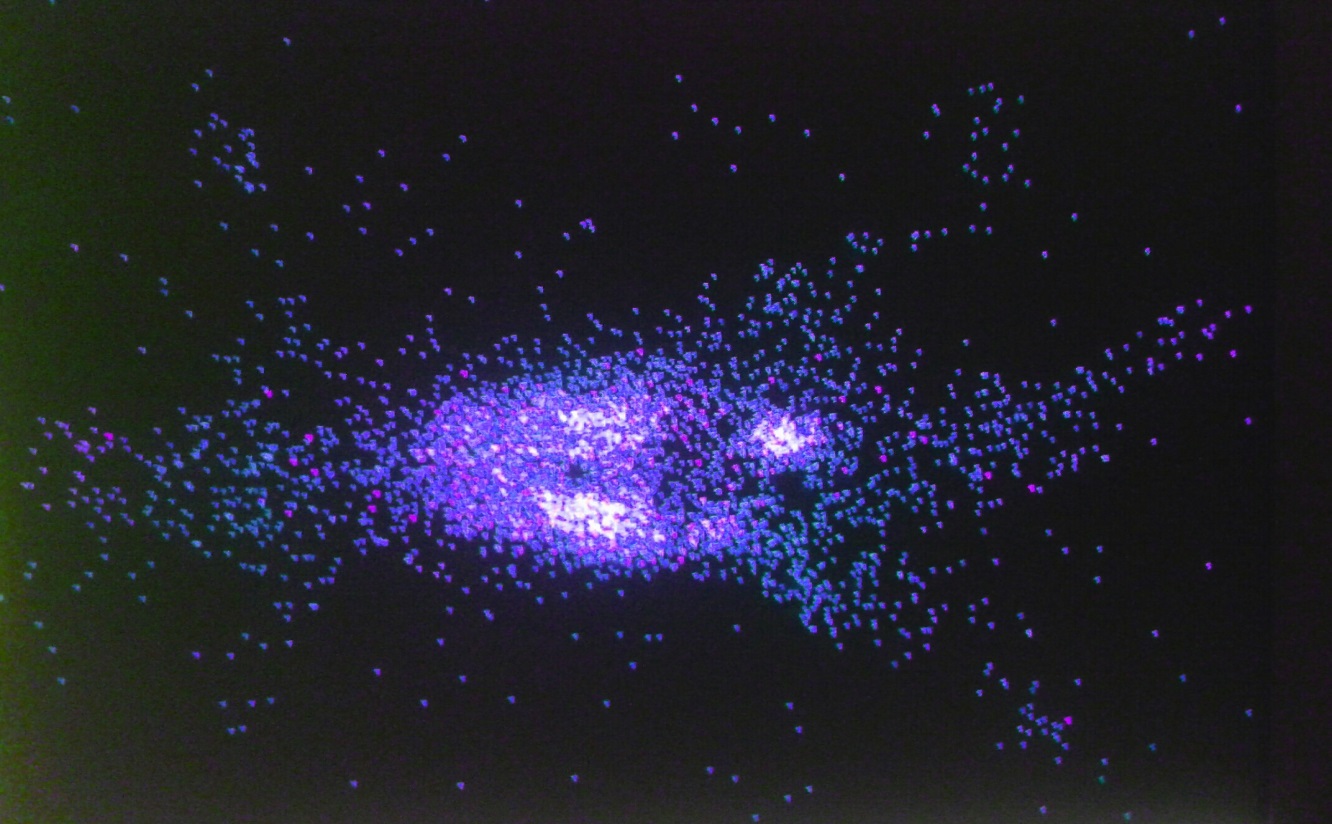


Supplementary Fig.S9. Gamma images of mice treated with ^99m^Tc-PEI/DNA complexes 2 h post injection.


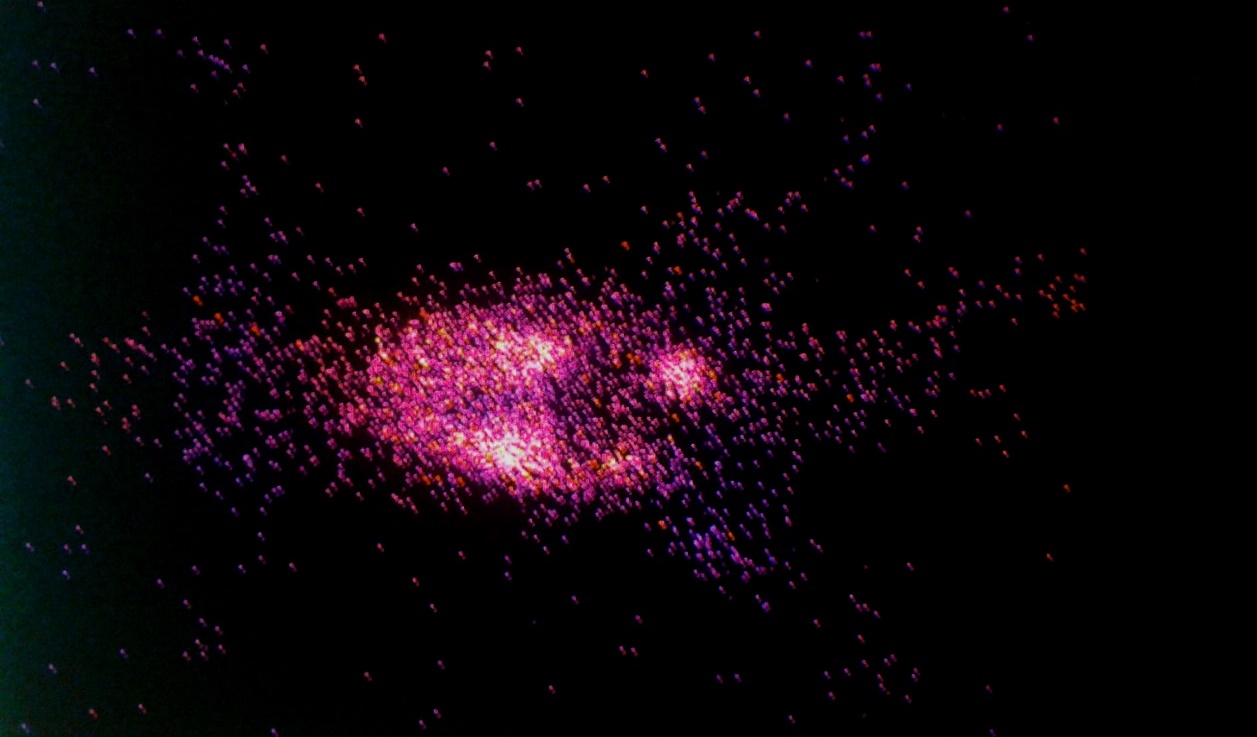


Supplementary Fig.S10. Gamma images of mice treated with ^99m^Tc-PEI/DNA complexes 4 h post injection.
